# Peripheral Retinal Neovascularization in a Patient with Sarcoidosis and Cocaine-Associated Autoimmunity

**DOI:** 10.1155/2021/9923260

**Published:** 2021-06-01

**Authors:** Ramak Roohipourmoallai, Samaneh Davoudi, Seyed Majid Hosseinian Benvidi, Siva S. R. Iyer

**Affiliations:** ^1^Department of Ophthalmology, University Of Florida, USA; ^2^Department of Internal Medicine, University of Florida, USA

## Abstract

A 63-year-old African-American female with history of sarcoidosis (lymph node biopsy proven) and cocaine abuse for 8 years was referred to us because of new floater. Her ocular history was unremarkable except for vague complaints of visual disturbance during a hospital admission in 2016. On presentation, her visual acuity was 20/400 in the right eye and 20/30 in the left eye. Dilated fundus exam and multimodal imaging showed thick epiretinal membrane (ERM) superior to optic nerve head causing a lamellar macular hole and intra retinal edema in the right eye, a full thickness macular hole, and peripheral neovascularization in the left eye. Peripheral vasculitis was appreciated in both eyes and peripheral neovascularization in the left eye on fluorescein angiography. The patient underwent laser therapy, and the new vessels regressed in the left eye without any changes in systemic medications. Multiple factors may contribute to retinal vasculitis and neovascularization including sarcoidosis, cocaine abuse, and other undiagnosed systemic vasculitis, which makes this case a mystery.

## 1. Introduction

There are many reported etiologies for retinal vasculitis as described by Rosenbaum et al. [1], including systemic disease or as a part of an infectious process. In view of the significant potential morbidity of these conditions, ophthalmologists need to be aware of these entities when faced with patients presenting with retinal vasculitis.

Sometimes, the definite diagnosis is a mystery specifically when there are multiple reasons for the same objective findings. Here, we report a case of proven history of sarcoidosis, cocaine abuse, and some nonspecific immunologic serologic findings in a patient with maculopathy, retinal vasculitis, and retinal neovascularization.

### 1.1. Investigation

A 63-year-old African-American female with a past medical history of lymph node biopsy proven sarcoidosis in her early 40s was referred to us for new left eye floater assessment. Her medical history included sarcoidosis (diagnosed in 2008), COPD, pulmonary hypertension, smoking, and crack/cocaine abuse for eight years. She had a history of many neurologic episodes (TIA) with no obvious evidence of acute intracranial abnormality, brain perfusion abnormalities, or flow-limiting stenosis on workup. She had several admissions to the hospital over the years due to chronic hypoxic respiratory failure. The patient had been on oral prednisone 50 mg daily, but she stopped taking it years prior to presentation because of adverse side effects of nightmares and hirsutism. Her ocular history was unremarkable except vague complaints of a visual disturbance during one of her hospital admissions in 2016.

On presentation, her visual acuity was 20/400 in the right eye and 20/30 in the left eye. On dilated exam, a dense epiretinal membrane (ERM) was seen in the superior macula extending from the optic nerve head and peripheral retinal neovascularization in the temporal retina.

### 1.2. Diagnosis

**Figure 1:**
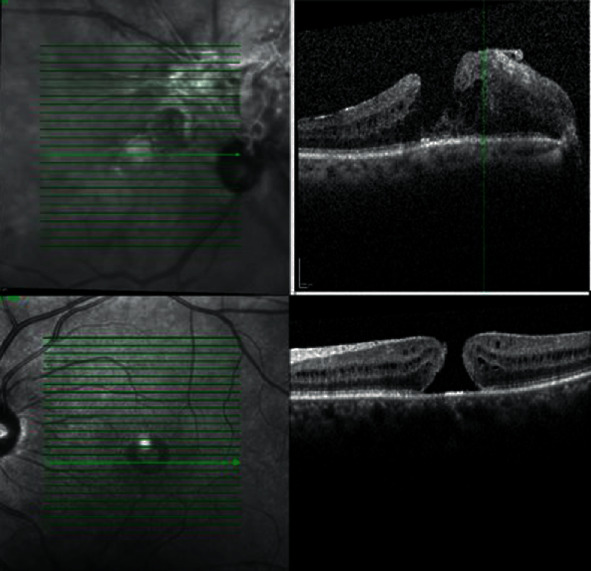
Macular OCT. Thick ERM on optic nerve head extending to the fovea causing lamellar macular hole and intra-retinal edema (OD). Full thickness macular hole (OS).

**Figure 2:**
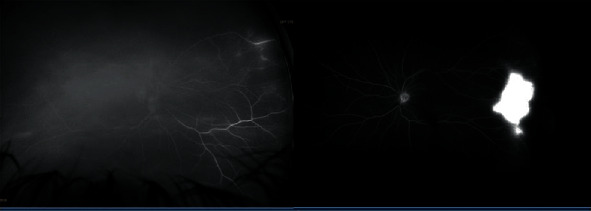
Fluorescein angiography showing peripheral vasculitis in the right eye and peripheral neovascularization in the left eye with significant leakage in temporal area.

Diagnostic testing including optical coherence tomography (OCT) showed a lamellar macular hole of the right eye and a full thickness macular hole in the left eye (Figure 1). Fluorescein angiography (FA) revealed peripheral vasculitis in both eyes in addition to a peripheral avascular area and temporal neovascularization in the left eye (Figure 2).

A review of systemic diagnostics included a chest CT six months prior that showed severe diffuse fibrosis bilaterally, emphysematous changes mostly prominently in the upper lobes along with cystic and honeycombing/reticulation changes. Her lab results were as follows: positive c-ANCA 1 : 320 (ref range < 1 : 20); positive PR3 41 (ref range 0-19 AU/ML); negative myeloperoxidase (MPO) Ab: 8 (ref range 0-19 AU/mL, negative angiotensin-converting enzyme (ACE): 26 (ref range 9-67 U/L), negative ANA and subsets DS DNA Ab (dsDNA) IgG < 1 : 10 (ref range < 1 : 10), negative ribonucleic protein (ENA) antibody 0 (ref range 0-40 AU/mL).

Peripheral retinal neovascularization could not be explained only with sarcoidosis alone, and another factor like cocaine use could be a contributing factor.

### 1.3. Treatment

**Figure 3:**
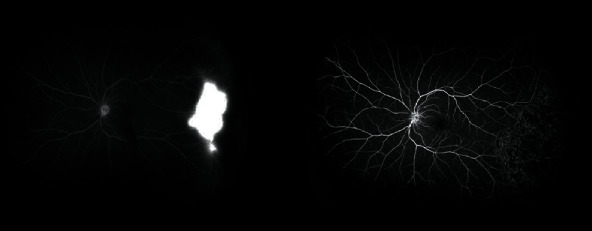
Neovascularization regressed after laser treatment in the left eye.

Retinal photocoagulation was performed in the avascular area, and the neovascularization regressed completely (Figure 3). As the patient did not complain about vision impairment, a surgical option was not pursued for the full thickness macular hole per patient request.

### 1.4. Follow-Up

Though the patient's peripheral retinal neovascularization was controlled with laser only, the patient was started on azathioprine by her treating team for her systemic issues.

## 2. Discussion

Here, we report a case of an African-American patient with a history of sarcoidosis and substance abuse (cocaine) who had vague visual symptoms on presentation and was found to have findings of vasculitis and peripheral avascular retina with neovascularization and macular abnormalities in both eyes. She had a history of acute floaters which was related to intermittent vitreous hemorrhage in the left eye due to neovascularization.

The eye findings of vasculitis, ERM, peripheral avascular area, and neovascularization can be explained with different medical issues including sarcoidosis, cocaine abuse, sickle cell, or a history of FEVR. The patient also had no history of ROP and was not a diabetic, eliminating those additional theoretical causes of retinal ischemia. Patient's medical history was only positive for sarcoidosis and cocaine abuse.

Sarcoidosis is a systemic multiorgan inflammatory disorder typically involving the lungs and lymphatics. The disease course may be variable in different patients. It has symptoms that can involve the respiratory system, extrathoracic specifically lymph nodes, skin, or eye and constitutional symptoms such as fatigue, weight loss, fever or night sweats, and erythema nodosum. 30-60% of patients have ophthalmic involvement. All ocular structures may be involved, but uveitis is the most frequent form of ocular manifestation, and up to 20–30% of sarcoidosis patients may be affected. In a retrospective series by Jamilloux et al. of proven (histologically) sarcoidosis; symptomatic uveitis affects 20–50% of patients [2].

There are intraocular signs suggestive of ocular sarcoidosis explained by Herbort et al. as follows [3]:

1. Mutton-fat keratic precipitates (large or small) and/or iris nodules (Koeppe/Busacca)
2. Trabecular meshwork nodules and/or tent-shaped peripheral anterior synechia
3. Snowballs/strings of pearls' vitreous opacities
4. Multiple chorioretinal peripheral lesions (active and/or atrophic)
5. Nodular and/or segmental periphlebitis (±candle wax drippings) and/or retinal macroaneurysm in an inflamed eye
6. Optic-disk nodule(s)/granuloma(s) and/or a solitary choroidal nodule
7. Bilateral inflammation (evident on clinical examination or on investigational imaging) Rothova et al. described sarcoid uveitis is usually bilateral (80–90%). Anterior uveitis is the most common anatomical form of intraocular inflammation (41–75% of sarcoid uveitis) followed by posterior, intermediate uveitis and panuveitis [4]

Our patient was diagnosed with vitreous hemorrhage due to peripheral neovascularization in the left eye which was the reason for having the new floaters. There are some ocular findings that are not compatible with sarcoidosis as the cause in this patient. She did not report any signs of anterior uveitis such as eye pain, photophobia, or redness. Isolated posterior segment involvement has been reported in only 5% of patients with sarcoid disease as explained by Pasadhika and Rosenbaum [5]. The most common posterior findings are chorioretinitis and retinal periphlebitis with retinal hemorrhage. We could see only mild peripheral periphlebitis, in addition to ERM and a macular hole which can be explained with uveitis as one of the reason for development of these surface abnormalities although our patient did not report any symptoms of previous acute uveitis.

Our patient had peripheral retinal neovascularization and abrupt peripheral vessels drop out which occur very rarely in sarcoidosis patients but have been reported with other accompanying disease such as sickle cell or talc retinopathy as explained by Tran [6]. Our patient did not have any history of sickle cell trait or disease, but had history of cocaine abuse which could have caused the peripheral retinal neovascularization, but not the vitreoretinal surface abnormalities.

Neovascularization of the peripheral retina can be present in a number of systemic and ocular diseases and it can also be manifested in drug abusers. Retinal vascular emboli such as talc are common in drug abusers; however, the only reason for aforementioned findings in this patient with specific history could be sarcoidosis in a cocaine abuse patient. The macular and optic nerve findings can be explained with chronic inflammation due to sarcoidosis but not cocaine abuse.

The patient responded to laser only without the addition of any systemic medication, which makes sarcoidosis less probable as the sole reason for peripheral retinal neovascularization. ACE was tested several times which was within normal limits. This normal test cannot exclude a sarcoidosis diagnosis as sarcoidosis was proven with positive lymph node biopsy but can correlate with disease inactivity. In a large series of patients with uveitis; ACE had a sensitivity of 84% and a specificity of 95% for the diagnosis of sarcoidosis. In this study by Baarsma et al., lysozyme was a less-sensitive (60%) and specific (76%) test [7].

The patient had a positive test for c-ANCA and PR3. Given this, granulomatosis with polyangiitis (GPA) may be considered; however, the patient did not have known upper airway or kidney involvement and her lung parenchyma abnormalities did not include some of the more classic manifestations of GPA [8] such as cavitation explained by Yates and Watts.

Retinal vasculitis can be seen in ANCA-associated vasculitis (AAV) [8], although the patient denied joint pain, swelling, skin rashes, sinus disease, and a history of hemoptysis or kidney disease. Lab studies ruled out excessive proteinuria and/or hematuria.

We think this patient's positive c-ANCA and PR3 positive tests were due to a history of cocaine use, which has been reported by Morcos and Lood et al. before in a cocaine-associated autoimmunity syndrome (CLAAS) [9]. This diagnosis can explain the peripheral neovascularization in this patient, which is not explained only with sarcoidosis.

Her vitreous hemorrhage has been controlled with sectoral angiographic-guided retinal photocoagulation, and she has not required escalation of systemic immunosuppression from an ocular standpoint.

## 3. Conclusion

Our patient with history of sarcoidosis developed peripheral vasculitis, peripheral retinal neovascularization, and vitreoretinal surface abnormalities that could not be explained with sarcoidosis only but could be explained with both sarcoidosis and her cocaine abuse and/or cocaine immune-related reaction.

## Data Availability

The data used to support the findings of this study are included within the article.

## References

[1] Rosenbaum J. T., Sibley C. H., Lin P. (2016). Retinal vasculitis. *Current Opinion in Rheumatology*.

[2] Jamilloux Y., Kodjikian L., Broussolle C., Sève P. (2014). Sarcoidosis and uveitis. *Autoimmunity Reviews*.

[3] Herbort C. P., Rao N. A., Mochizuki M., the members of the Scientific Commi (2009). International criteria for the diagnosis of ocular sarcoidosis: results of the first International Workshop On Ocular Sarcoidosis (IWOS). *Ocular Immunology and Inflammation*.

[4] Rothova A., Alberts C., Glasius E., Kijlstra A., Buitenhuis H. J., Breebaart A. C. (1989). Risk factors for ocular sarcoidosis. *Documenta Ophthalmologica*.

[5] Pasadhika S., Rosenbaum J. T. (2015). Ocular sarcoidosis. *Clinics in Chest Medicine*.

[6] Tran K. H., Ilsen P. F. (2007). Peripheral retinal neovascularization in talc retinopathy. *Optometry - Journal of the American Optometric Association*.

[7] Baarsma G. S., La Hey E., Glasius E., de Vries J., Kijlstra A. (1987). The predictive value of serum angiotensin converting enzyme and lysozyme levels in the diagnosis of ocular sarcoidosis. *American Journal of Ophthalmology*.

[8] Yates M., Watts R. (2017). ANCA-associated vasculitis. *Clinical Medicine (London, England)*.

[9] Morcos M. B., Lood C., Hughes G. C. (2019). Demographic, clinical, and immunologic correlates among a cohort of 50 cocaine users demonstrating antineutrophil cytoplasmic antibodies. *The Journal of Rheumatology*.

